# Nanoreactor Design Based on Self-Assembling Protein Nanocages

**DOI:** 10.3390/ijms20030592

**Published:** 2019-01-30

**Authors:** Huimei Ren, Shaozhou Zhu, Guojun Zheng

**Affiliations:** 1 Center for Drug Evaluation (CDE), National Medical Products Administration (NMPA), Beijing 100022, China; renhm@cde.org.cn; 2 State Key Laboratory of Chemical Resources Engineering, Beijing University of Chemical Technology, Beijing 100029, China; 3 College of Life Science and Technology, Beijing University of Chemical Technology, Beijing 100029, China

**Keywords:** nanoreactor, virus capsids, encapsulins, artificial protein dodecahedron, self-assembling, biocatalysis, biosynthesis, synthetic biology

## Abstract

Self-assembling proteins that form diverse architectures are widely used in material science and nanobiotechnology. One class belongs to protein nanocages, which are compartments with nanosized internal spaces. Because of the precise nanoscale structures, proteinaceous compartments are ideal materials for use as general platforms to create distinct microenvironments within confined cellular environments. This spatial organization strategy brings several advantages including the protection of catalyst cargo, faster turnover rates, and avoiding side reactions. Inspired by diverse molecular machines in nature, bioengineers have developed a variety of self-assembling supramolecular protein cages for use as biosynthetic nanoreactors that mimic natural systems. In this mini-review, we summarize current progress and ongoing efforts creating self-assembling protein based nanoreactors and their use in biocatalysis and synthetic biology. We also highlight the prospects for future research on these versatile nanomaterials.

## 1. Introduction

Subcellular organization is an essential strategy for cells to orchestrate important cellular processes and is believed to be a general feature of life [1,2,3,4]. While eukaryotes are well known to organize their cellular interiors with diverse membrane-bound organelles such as chloroplasts, Golgi bodies, and lysosomes [5,6,7], current research on microorganisms has drawn an unexpected picture of bacterial cells where a myriad of subcellular structures were developed by the evolution of self-assembling proteinaceous microcompartments [8,9,10,11,12,13,14,15,16,17]. Organelles and the bacterial microcompartments create a unique spatial segregation allowing sequestration of specific proteins and metabolic pathways [10,12,13,17]. This strategy allows for several advantages, e.g., (a) increasing the efficiency of the sequestered biosynthetic pathways; (b) enrichment of the substrates and products; and (c) unique microenvironments for unstable catalysts [14,15,16].

**Figure 1:**
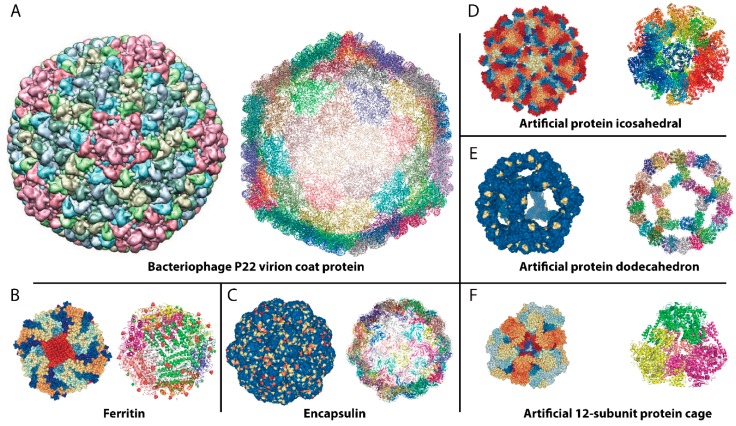
Examples of natural and non-natural proteinaceous compartments. Structures are shown: (**A**), Bacteriophage P22, (PDB: 2XYZ); (**B**), Ferritin, (PDB: 6A4U); (**C**), Encapsulins, (PDB: 4PT2); (**D**), Artificial protein icosahedral, (PDB: 5KP9); (**E**), Artificial protein dodecahedron, (PDB: 5IM5); (**F**), Artificial 12-subunit protein cage, (PDB: 3VDX).

Compared with membrane-bound organelles developed by eukaryotic cells, proteinaceous microcompartments with predictable architectures are of great interest to bioengineers due to their robust self-assembly properties, solubility, and biocompatibility [17]. Moreover, their structures are “programmable” for broad application in nanobiotechnology and chemistry, as well as functional materials [11]. A variety of naturally occurring proteinaceous microcompartments have been reported, including viral capsids (Figure 1A) [18,19,20], ferritin (Figure 1B) [21,22,23,24], encapsulins (Figure 1C) [8,25,26,27], carboxysomes [12,28,29,30,31], and 1,2-propanediol utilization (Pdu) or ethanolamine utilization (Eut) microcompartments [32,33,34,35,36,37]. Apart from the naturally occurring microcompartments, several artificial proteinaceous microcompartments have been created recently. These include a 60-subunit protein dodecahedron protein nanocage (Figure 1D) [38], several 120-subunit two-component icosahedral protein nanocages (Figure 1E), and a self-assembled 600-kDa protein homododecamer cage (Figure 1F) [39,40]. These highly symmetric self-assembled protein architectures have an empty interior and can create microcompartments ranging in size from 10 to 500 nm (diameter). Notably, the rationally designed proteinaceous microcompartments have several superior properties [38,39,40]. Compared to naturally occurring ones, the artificial systems are precisely designed to make them accessible. Therefore, these fascinating scaffolds provide toolboxes for nanoreactor designs where enzymes can be sequestered in the interior, allowing the microcompartments to provide microenvironments similar to naturally occurring organelles [11,37].

In recent years, bioengineers have begun to utilize the advantages of nanocages by manipulating their structures. Both top-down and bottom-up approaches have been developed to construct nanoreactors for biocatalysis and synthetic biology [41,42,43]. Several proteinaceous microcompartments have been engineered to load exogenous biocatalysts and have been successfully constructed in well-studied hosts like *Escherichia coli* [11,44]. These artificial organelles provide a new platform to design a generation of nanofactories for synthetic biology. Moreover, with the development of novel discovery strategies such as genome mining and computer-aided design methods, plenty of novel proteinaceous microcompartments have been discovered and created that offer tremendous opportunities to design new catalytic nanoreactors [26,38,39]. In this mini-review, we highlight recent significant achievements in the design and construction of catalytic nanoreactors for biocatalysis based on self-assembling supramolecular protein complexes. A select number of proteinaceous microcompartments and the strategies used to construct the nanoreactors will be presented together with their applications.

## 2. Virus Capsids

During natural evolution, viruses have developed a large variety of capsids to package, protect, and deliver their DNA or RNA genome [18,19,20]. The well-defined 3D structures make them perfect building blocks for nanobiotechnology applications [45]. Generally, they are protein-based microcompartments of about 15–500 nm (diameter) overall with variable interior spaces [46,47]. For example, the bacteriophages P22 forms an icosahedral cage with an outer diameter of about 64 nm that self-assembles from 420 copies of a single capsid protein [48]. The capsid proteins are easily prepared by heterologous expression and then functionalized [49]. Recent years have realized a large number of applications for these capsids in nanobiotechnology including hybrid nanostructured materials [50,51,52], vaccine development [53,54,55], medicine delivery platforms, and biomineralization systems [54,56,57,58]. In recent reports, nanoreactors based on viral capsid containers have been developed where enzymes are loaded before assembly [59].

Several viral capsids have been selected as models for reaction vessels including Cowpea Chlorotic Mottle Virus (CCMV), and bacteriophages P22, Qβ, and MS2 [60,61,62,63,64,65,66,67]. To successfully assemble the biocatalyst cargo into the nanocontainer, a guide tag is necessary for spatial arrangement. This has been accomplished by using peptide tags, coiled-coil helical interactions, DNA tags, or RNA tags to mediate enzymatic cargoes packing into protein cages [60,61,62,63,64,65,66,67].

**Figure 2:**
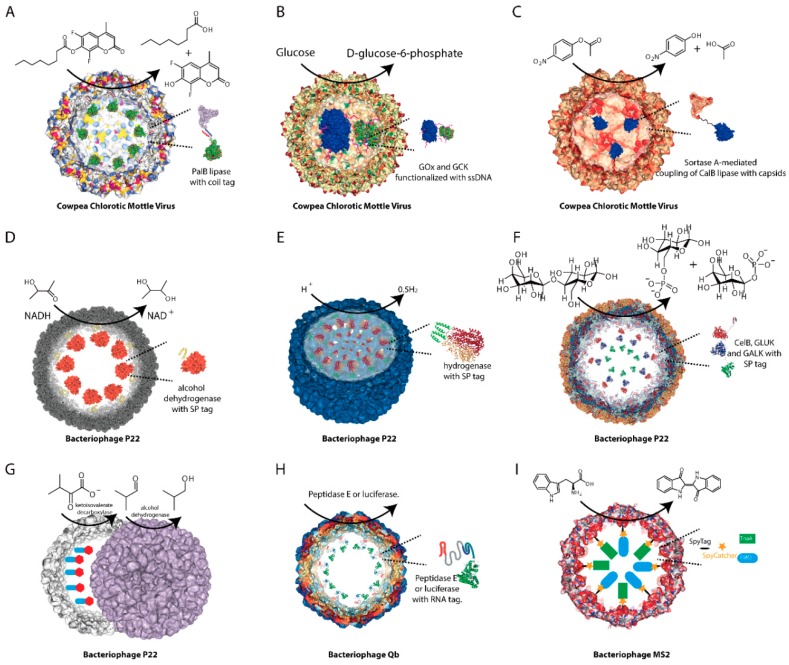
Examples of viral capsids as nanocontainers for nanoreactor construction.

The first virus-based enzyme nanoreactor was reported in 2007 in which horseradish peroxidase was successfully loaded into the capsids of CCMV [59]. Later, Cornelissen et al. developed strategies to position specific enzymes inside a virus capsid and constructed several nanoreactors based on CCMV [66,67,68,69]. By using heterodimeric coiled-coil peptide oligomers, cargo proteins fused with these oligomers could be spontaneously assembled within capsids modified with a compatible coil peptide oligomer [67,68,69]. As proof of concept, an enhanced green fluorescent protein (EGFP) was encapsulated in a controlled manner [67]. Subsequently, lipase B from *Pseudozyma antarctica* and EGFP were precisely loaded into the CCMV capsid by the same strategy, marking successful construction of a nanoreactor (Figure 2A) [69]. Compared to free enzymes, the nanoreactors with encapsulated cargoes showed an increased overall reaction rate, which was nearly independent of the number of enzymes it sequestered [68,69].

Another packing strategy developed by the same group utilized nucleic acid tags with negative charges [66]. Generally, the cargo proteins were chemically coupled with single-stranded DNA (ssDNA) or its complementary sequence (csDNA). The resulting protein–DNA hybridized complexes were readily encapsulated via non-covalent interaction, and several nanoreactors were constructed this way [66]. For example, glucose oxidase (GOx) and gluconokinase (GCK) were successfully co-encapsulated and the enzymatic cascade system was used to produce ribulose-5-phosphate from glucose (Figure 2B) [66]. This work provided a general method for co-encapsulation of enzymes, which greatly enhances metabolic efficiency. In addition to these successes, van Hest et al. also developed several nanoreactors based on CCMV using a different method [65,70]. They found that Sortase A could be used as a ligase to link cargo enzymes to the glycine-tagged N-termini of CCMV capsids [65,70]. Cargo enzymes with a C-terminal LPETG-motif are easily packed into the capsids in a way that is minimally disruptive to the cargo. With this strategy, an industrial biocatalyst CalB lipase was successfully encapsulated and lead to a lipase based nanoreactor (Figure 2C) [65,70].

Bacteriophage P22 is another versatile platform for nanoreactor development [49,62,63,64,71]. Unlike CCMV, the coat protein (CP) of Bacteriophage P22 can assemble into a T = 7 icosahedral capsid with the aid of hundreds of copies of scaffolding protein (SP) [72,73]. Past research has shown that the C terminus of the scaffolding protein (SP tag) is essential and sufficient for the capsid assembly [64,72,73]. By fusing an SP tag to the cargo enzymes, Douglas et al. developed nanoreactors for biocatalysis and synthetic biology [49,62,63,64,71]. These include an alcohol dehydrogenase AdhD based nanoreactor (Figure 2D) [49], [NiFe]-hydrogenase based self-assembling biomolecular catalysts for hydrogen production (Figure 2E) [63], and a three-enzyme cascade nanoreactor for lactose metabolism (Figure 2F) [64,71]. Notably, the capsid provides stability and protection to the cargo enzymes in all cases [49,64,71]. Very recently, they expanded the application of Bacteriophage P22 capsid to construct three-dimensional superlattice catalysts [62]. A ketoisovalerate decarboxylase (KivD) and an alcohol dehydrogenase A (AdhA) were encapsulated within the P22 capsids separately, and these materials were then spontaneously self-assembled into higher ordered superlattice materials with the assistance of positively charged PAMAM dendrimers (Figure 2G) [62]. The resulting superlattice catalysts could be used to perform an enzymatic cascade reaction for synthesis of isobutanol and showed several superior properties including accelerated catalytic efficiency, and they were easily recovered and recycled [62].

RNA viruses have also been selected as reaction vessels. Recently, Bacteriophage Qβ was used to package functional enzymes using protein–RNA interactions [61]. The RNA genome of Bacteriophage Qβ forms a hairpin structure, which has a high-affinity interaction with the interior-facing residues of the CP and can be used to mediate the encapsulation of cargo enzymes [61,74]. To facilitate RNA-directed nanoreactor construction, an RNA aptamer specifically binding to an arginine-rich peptide (Rev) derived from HIV-1 was introduced into the CP mRNA [61]. Two cargo enzymes (Peptidase E and luciferase) fused with Rev Tags were then then successfully packed into Qβ particles (Figure 2H) [61].

Manipulation of SpyTag/SpyCatcher is another way to mediate cargo encapsulation [60,75]. Recently, Giessen et al. developed a catalytic nanoreactor based on an engineered Bacteriophage M2 capsid [75]. The phage MS2 capsid protein was engineered to display the SPY tags facing the interior. By introducing a SPY catcher tag onto the cargo enzymes, catalysts were then spontaneously cross-linked with the interior surface of the capsid [60,75]. As proof of concept, two active enzymes for indigo biosynthesis were successfully targeted to the engineered capsids (Figure 2I) [75]. In vivo studies showed that the nanoreactor improved indigo production efficiency relative to unencapsulated enzymes by 60%. Moreover, in vitro studies showed that enzymes packaged in purified nanoreactors show enhanced long-term stability compared to free enzymes [75].

## 3. Encapsulins

Encapsulins are a new family of microbial proteinaceous compartments that have been engineered for nanoreactor construction [76,77]. Typically, encapsulin has an overall size of about 20–40 nm (diameter), which is very similar to virus [27,78,79,80,81]. Structural studies have showed that encapsulins can be generally classified into types T = 1 (60 subunits, 20–24 nm) and T = 3 (180 subunits, 30–32 nm) hollow icosahedral capsids [25,82,83,84,85,86,87,88]. Interestingly, a very recent genome mining study revealed that encapsulins are widely distributed in nature [26]. Up to 900 putative encapsulin systems in diverse bacterial and archaeal genomes have been discovered by in silico analysis, which provides an array of nanoplates for biomedicine, nanobiotechnology, and materials science [26]. Indeed, encapsulins have already been engineered as a scaffold for targeted diagnostics and therapeutic delivery systems, a nanocontainer for metal nanoparticles, and very recently, protein containers for nanoreactor construction [27,76,77,78,79,86]. In fact, encapsulins are naturally occurring nanoreactors that encapsulate specific cargo proteins and are involved in diverse cell processes including iron mineralization, oxidative and nitrosative stress resistance, and anaerobic ammonium oxidation [8,26,76,82,85]. Thus far, more than 10 different types of cargo proteins have been identified. Most of them have a specific terminal tag that mediates the packaging of the cargo enzymes inside the protein shells [8,26,76,82,85].

**Figure 3:**
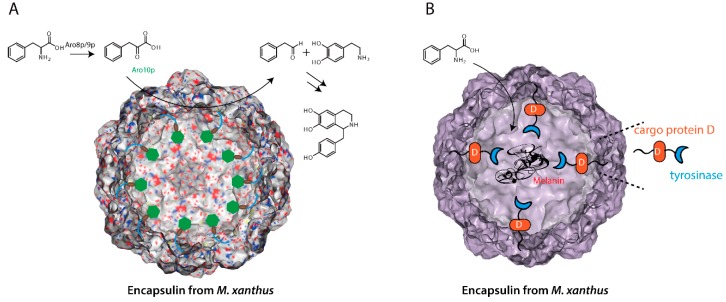
Examples of Encapsulins as nanocontainers for nanoreactor construction.

Mimicking the concept of natural functional encapsulins, two artificial nanoreactors were successfully constructed based on the encapsulin system from *Myxococcus xanthus* [76,77]. Silver et al. successfully expressed the prokaryotic encapsulin system in the eukaryotic yeast *Saccharomyces cerevisiae* [76]. In the native system, three cargo proteins are simultaneously packed into the capsids mediated by short targeting peptides (TPs) located at the C termini of the cargo enzymes [25]. By fusing TP tags to the heterologous proteins, different enzymes were able to be selectively encapsulated [76]. Specifically, a tetrameric pyruvate decarboxylase enzyme (Aro10p) was selected and a nanoreactor for the biosynthesis of 4-hydroxyphenylacetaldehyde (4-HPAA) was constructed (Figure 3A) [76]. This example demonstrates that encapsulin compartments could be selected as a general platform for organelle construction in eukaryotes and has potential for wide application in synthetic biology.

Independently, Westmeyer et al. showed that engineered encapsulin from *M. xanthus* could be produced in mammalian cells [77]. Moreover, various non-natural cargo could be self-targeted and encapsulated inside the shell proteins using the target peptides [77]. This demonstrates that the encapsulin system could also be developed as a nanoreactor chamber within mammalian cells [77]. For example, the split luciferase parts LgBit and SmBit were separately fused to the native C and B proteins and functional luciferase activity was observed upon packaging [77]. Moreover, an active tyrosinase from *Bacillus megaterium* was fused to the native cargo D protein and an artificial melanosome was successfully constructed that was readily detected by robust multispectral optoacoustic tomography (MSOT) based on the production of toxic melanin in the nanoshells (Figure 3B) [77]. Apart from these applications, the iron-loading encapsulins are also outstanding reporters for electron microscopy (EM) [77]. These studies prove that encapsulins might have a wide application for eukaryotic cell engineering, optical imaging, and emerging cell therapies.

## 4. Artificial Protein Dodecahedron

**Figure 4:**
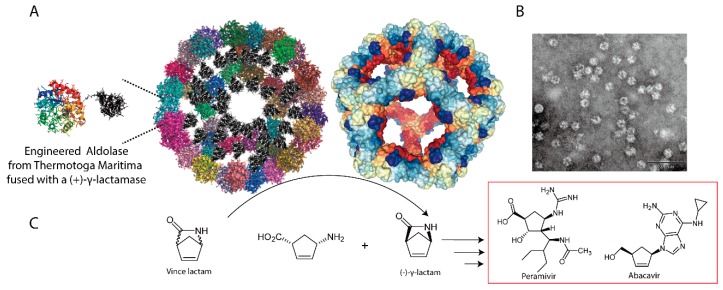
Examples of artificial protein dodecahedron as nanocontainers for nanoreactor construction. (**A**) Self-assembly of γ-lactamase nanoreactor based on engineered aldolase from *Thermotoga maritima* (**B**) Structures of constructed nanoreactor, representative images from high-resolution transmission electron microscopy (TEM) (**C**) Scheme showing the reaction catalyzed by the nanoreactor.

The rapid development of supercomputing and bioinformatics technology has lead us to a new age of de novo protein design [89,90,91,92,93,94]. Following the basic physicochemical principles that direct protein folding, computational biochemists are now able to design a wide range of intriguing structures with atomic-level accuracy [89,90,91,92,93]. One of the more interesting de novo protein designs was the creation of self-assembling protein nanocages [38,39,40]. For example, Baker et al. have designed one- and two-component protein nanocages with dodecahedral or icosahedral symmetry using 60 or 120 subunits by modifying the interfaces between proteins [38,39]. These high-symmetry artificial protein nanomaterials have large interior volumes and are widely applicable in vaccine development and synthetic biology. We have recently demonstrated that the artificial hypersTable 60-subunit protein dodecahedron could be functionalized as a scaffold for nanoreactor construction [95]. The engineered trimeric aldolase from *Thermotoga maritima* with a modified interface was fused with an industrial biocatalyst (+)-γ-lactamase from *Microbacterium hydrocarbonoxydans* and the hybridized protein could be self-assembled into an organelle-like nanodevice (Figure 4) [95]. The constructed nanoreactor is readily used for enzymatic resolution of Vince lactam, an important intermediate for synthesis of carbocyclic nucleoside medicines [95,96,97]. Notably, the designed nanoreactors could confer a significant benefit to the biocatalyst cargo. The encapsulated (+)-γ-lactamase exhibits significantly improved stabilities with respect to heat, organic solvent, and protease degradation. Moreover, it shows better substrate tolerance than the free enzyme [95]. This research demonstrates that bio-designed artificial protein nanocages are an effective way to improve the stability and strength of biocatalysts and might have broader applications in sustainable catalysis and synthetic biology.

## 5. Conclusions and Outlook

Biological systems have evolved various proteinaceous nanocompartments to sustain important life processes [8,14,15,17,26,27]. These robust molecular constructs are generally formed by self-assembly and have inspired the creation of diverse artificial protein nanocages [11,12,13,16,24]. Recent advances in understanding these nanomaterials have led to the construction of synthetic nano-biological devices [64,65,70,72,75]. Generally, the main benefit in coupling nanomaterials with catalytic reactions is to bring them to an appropriate nanoscale (20–50 nm diameter). They are highly homogeneous, easily prepared, and chemically or genetically functionalized [66,67,71,72,95]. Moreover, the transition of the assembly–depolymerization state of these nanocages can be precisely controlled, which makes them an important class of biological nano-elements that can be used to construct multi-functional nano-structures and devices [49,61,63,64].

In this review, we have summarized recent achievements in nanoreactor design based on self-assembling protein nanocages for biocatalysis and synthetic biology. Theoretically, by loading the cargo enzymes into the protein nanocages, a limited reaction space can be provided to control the entry and exit of substrate and product, thereby facilitating the regulation of the catalytic reaction [49,61,63,64,75]. Viral capsids are by far the most commonly used material for nanoreactor construction [49,61,63,64,67,68]. Recent progress on encapsulin and artificial protein nanocages provide more options and are ripe for expansion in the near future [8,38,39,89]. It has been proven that these self-assembled nanomaterials could confer benefits by serving as catalytic reaction vessels to the biocatalyst cargo [49,64,71,75,95]. Despite these achievements, there are still several directions that warrant continued research. First, most of the current research only provides proof of concept. These newer nanomaterials have not been widely applied in industrial biocatalysis or synthetic biology. This issue needs to be addressed with more industrial biocatalyst systems. With scaled applications, nanoreactor construction could become an invaluable device for all biocatalysis processes in the future.

Another interesting area for future study is the development of more complex metabolic pathways in nanoreactors. Thus far, only up to three different kinds of cargo enzymes have been sequestered into a single nanoreactor [71]. Developing more complex nanoreactors could significantly expand the application of proteinaceous microcompartments in synthetic biology. This direction is very promising since more complex encapsulin systems have been discovered and more complicated artificial protein nanocages have been designed [26,39]. For example, Giessen et al. proposed that encapsulins might be proper candidates for construction of an artificial carboxysome [98]. These areas still need to be investigated.

In summary, we expect that nanoreactors based on proteinaceous microcompartments will find widespread applications in biocatalysis and synthetic biology. We envision that with the accumulation of understanding on newly discovered and created self-assembling protein nanocage systems as well as the development of new toolboxes for protein engineering, diverse unprecedented nanoreactors will be created for industrial biocatalysis. Ideally, these artificial molecular machines will be widely applicable in novel nano-factory construction and will promote the development of green biochemical processes in the industry.

## References

[1] Greene S.E., Komeili A. (2012). Biogenesis and subcellular organization of the magnetosome organelles of magnetotactic bacteria. Curr. Opin. Cell Biol..

[2] Gut G., Herrmann M.D., Pelkmans L. (2018). Multiplexed protein maps link subcellular organization to cellular states. Science.

[3] Gingras A.C., Abe K.T., Raught B. (2018). Getting to know the neighborhood: Using proximity-dependent biotinylation to characterize protein complexes and map organelles. Curr. Opin. Chem. Biol..

[4] Surovtsev I.V., Jacobs-Wagner C. (2018). Subcellular Organization: A Critical Feature of Bacterial Cell Replication. Cell.

[5] Carraretto L., Teardo E., Checchetto V., Finazzi G., Uozumi N., Szabo I. (2016). Ion Channels in Plant Bioenergetic Organelles, Chloroplasts and Mitochondria: From Molecular Identification to Function. Mol. Plant.

[6] Luzio J.P., Pryor P.R., Bright N.A. (2007). Lysosomes: Fusion and function. Nat. Rev. Mol. Cell Biol..

[7] Martin S., Parton R.G. (2006). Lipid droplets: A unified view of a dynamic organelle. Nat. Rev. Mol. Cell Biol..

[8] Nichols R.J., Cassidy-Amstutz C., Chaijarasphong T., Savage D.F. (2017). Encapsulins: Molecular biology of the shell. Crit. Rev. Biochem. Mol. Biol..

[9] Saier M.H. (2013). Microcompartments and protein machines in prokaryotes. J. Mol. Microbiol. Biotechnol..

[10] Frank S., Lawrence A.D., Prentice M.B., Warren M.J. (2013). Bacterial microcompartments moving into a synthetic biological world. J. Biotechnol..

[11] Giessen T.W., Silver P.A. (2016). Encapsulation as a Strategy for the Design of Biological Compartmentalization. J. Mol. Biol..

[12] Rae B.D., Long B.M., Whitehead L.F., Forster B., Badger M.R., Price G.D. (2013). Cyanobacterial carboxysomes: Microcompartments that facilitate CO_2_ fixation. J. Mol. Microbiol. Biotechnol..

[13] Cheng S., Liu Y., Crowley C.S., Yeates T.O., Bobik T.A. (2008). Bacterial microcompartments: Their properties and paradoxes. BioEssays.

[14] Rae B.D., Long B.M., Badger M.R., Price G.D. (2013). Functions, compositions, and evolution of the two types of carboxysomes: Polyhedral microcompartments that facilitate CO_2_ fixation in cyanobacteria and some proteobacteria. Microbiol. Mol. Biol. Rev..

[15] Yeates T.O., Jorda J., Bobik T.A. (2013). The shells of BMC-type microcompartment organelles in bacteria. J. Mol. Microbiol. Biotechnol..

[16] O’Connell J.D., Zhao A., Ellington A.D., Marcotte E.M. (2012). Dynamic reorganization of metabolic enzymes into intracellular bodies. Annu. Rev. Cell Dev. Biol..

[17] Lee M.J., Palmer D.J., Warren M.J. (2018). Biotechnological Advances in Bacterial Microcompartment Technology. Trends Biotechnol..

[18] Mateu M.G. (2016). Assembly, Engineering and Applications of Virus-Based Protein Nanoparticles. Adv. Exp. Med. Biol..

[19] Mateu M.G. (2013). Assembly, stability and dynamics of virus capsids. Arch. Biochem. Biophys..

[20] Perlmutter J.D., Hagan M.F. (2015). Mechanisms of virus assembly. Annu. Rev. Phys. Chem..

[21] Theil E.C. (2013). Ferritin: The protein nanocage and iron biomineral in health and in disease. Inorg. Chem..

[22] Zang J., Chen H., Zhao G., Wang F., Ren F. (2017). Ferritin cage for encapsulation and delivery of bioactive nutrients: From structure, property to applications. Crit. Rev. Food Sci. Nutr..

[23] Hagen W.R., Hagedoorn P.L., Honarmand Ebrahimi K. (2017). The workings of ferritin: A crossroad of opinions. Metallomics.

[24] Arosio P., Elia L., Poli M. (2017). Ferritin, cellular iron storage and regulation. IUBMB Life.

[25] McHugh C.A., Fontana J., Nemecek D., Cheng N., Aksyuk A.A., Heymann J.B., Winkler D.C., Lam A.S., Wall J.S., Steven A.C. (2014). A virus capsid-like nanocompartment that stores iron and protects bacteria from oxidative stress. EMBO J..

[26] Giessen T.W., Silver P.A. (2017). Widespread distribution of encapsulin nanocompartments reveals functional diversity. Nat. Microbiol..

[27] Giessen T.W. (2016). Encapsulins: Microbial nanocompartments with applications in biomedicine, nanobiotechnology and materials science. Curr. Opin. Chem. Biol..

[28] Kerfeld C.A., Melnicki M.R. (2016). Assembly, function and evolution of cyanobacterial carboxysomes. Curr. Opin. Plant Biol..

[29] Yeates T.O., Tsai Y., Tanaka S., Sawaya M.R., Kerfeld C.A. (2007). Self-assembly in the carboxysome: A viral capsid-like protein shell in bacterial cells. Biochem. Soc. Trans..

[30] Turmo A., Gonzalez-Esquer C.R., Kerfeld C.A. (2017). Carboxysomes: Metabolic modules for CO_2_ fixation. FEMS Microbiol. Lett..

[31] Yeates T.O., Kerfeld C.A., Heinhorst S., Cannon G.C., Shively J.M. (2008). Protein-based organelles in bacteria: Carboxysomes and related microcompartments. Nat. Rev. Microbiol..

[32] Tocheva E.I., Matson E.G., Cheng S.N., Chen W.G., Leadbetter J.R., Jensen G.J. (2014). Structure and expression of propanediol utilization microcompartments in Acetonema longum. J. Bacteriol..

[33] Fan C., Cheng S., Sinha S., Bobik T.A. (2012). Interactions between the termini of lumen enzymes and shell proteins mediate enzyme encapsulation into bacterial microcompartments. Proc. Natl. Acad. Sci. USA.

[34] Fan C., Cheng S., Liu Y., Escobar C.M., Crowley C.S., Jefferson R.E., Yeates T.O., Bobik T.A. (2010). Short N-terminal sequences package proteins into bacterial microcompartments. Proc. Natl. Acad. Sci. USA.

[35] Held M., Quin M.B., Schmidt-Dannert C. (2013). Eut bacterial microcompartments: Insights into their function, structure, and bioengineering applications. J. Mol. Microbiol. Biotechnol..

[36] Tanaka S., Sawaya M.R., Yeates T.O. (2010). Structure and mechanisms of a protein-based organelle in Escherichia coli. Science.

[37] Quin M.B., Perdue S.A., Hsu S.Y., Schmidt-Dannert C. (2016). Encapsulation of multiple cargo proteins within recombinant Eut nanocompartments. Appl. Microbiol. Biotechnol..

[38] Hsia Y., Bale J.B., Gonen S., Shi D., Sheffler W., Fong K.K., Nattermann U., Xu C., Huang P.S., Ravichandran R. (2016). Design of a hypersTable 60-subunit protein dodecahedron. [corrected]. Nature.

[39] Bale J.B., Gonen S., Liu Y., Sheffler W., Ellis D., Thomas C., Cascio D., Yeates T.O., Gonen T., King N.P. (2016). Accurate design of megadalton-scale two-component icosahedral protein complexes. Science.

[40] Lai Y.T., Cascio D., Yeates T.O. (2012). Structure of a 16-nm cage designed by using protein oligomers. Science.

[41] Jakobson C.M., Slininger Lee M.F., Tullman-Ercek D. (2017). De novo design of signal sequences to localize cargo to the 1,2-propanediol utilization microcompartment. Protein Sci..

[42] Schoonen L., van Hest J.C. (2016). Compartmentalization Approaches in Soft Matter Science: From Nanoreactor Development to Organelle Mimics. Adv. Mater..

[43] Gonzalez-Esquer C.R., Newnham S.E., Kerfeld C.A. (2016). Bacterial microcompartments as metabolic modules for plant synthetic biology. Plant J..

[44] Maity B., Fujita K., Ueno T. (2015). Use of the confined spaces of apo-ferritin and virus capsids as nanoreactors for catalytic reactions. Curr. Opin. Chem. Biol..

[45] Bajaj S., Banerjee M. (2015). Engineering Virus Capsids Into Biomedical Delivery Vehicles: Structural Engineering Problems in Nanoscale. J. Biomed. Nanotechnol..

[46] Douglas T., Young M. (2006). Viruses: Making friends with old foes. Science.

[47] Ludwig C., Wagner R. (2007). Virus-like particles-universal molecular toolboxes. Curr. Opin. Biotechnol..

[48] Johnson J.E., Chiu W. (2007). DNA packaging and delivery machines in tailed bacteriophages. Curr. Opin. Struct. Biol..

[49] Patterson D.P., Prevelige P.E., Douglas T. (2012). Nanoreactors by programmed enzyme encapsulation inside the capsid of the bacteriophage P22. ACS Nano.

[50] Soto C.M., Ratna B.R. (2010). Virus hybrids as nanomaterials for biotechnology. Curr. Opin. Biotechnol..

[51] Nam K.T., Kim D.W., Yoo P.J., Chiang C.Y., Meethong N., Hammond P.T., Chiang Y.M., Belcher A.M. (2006). Virus-enabled synthesis and assembly of nanowires for lithium ion battery electrodes. Science.

[52] Mann S. (2009). Self-assembly and transformation of hybrid nano-objects and nanostructures under equilibrium and non-equilibrium conditions. Nat. Mater..

[53] Kratz P.A., Bottcher B., Nassal M. (1999). Native display of complete foreign protein domains on the surface of hepatitis B virus capsids. Proc. Natl. Acad. Sci. USA.

[54] Garcea R.L., Gissmann L. (2004). Virus-like particles as vaccines and vessels for the delivery of small molecules. Curr. Opin. Biotechnol..

[55] Noad R., Roy P. (2003). Virus-like particles as immunogens. Trends Microbiol..

[56] Ma Y., Nolte R.J., Cornelissen J.J. (2012). Virus-based nanocarriers for drug delivery. Adv. Drug Deliv. Rev..

[57] Stephanopoulos N., Tong G.J., Hsiao S.C., Francis M.B. (2010). Dual-surface modified virus capsids for targeted delivery of photodynamic agents to cancer cells. ACS Nano.

[58] Liu Z., Qiao J., Niu Z., Wang Q. (2012). Natural supramolecular building blocks: From virus coat proteins to viral nanoparticles. Chem. Soc. Rev..

[59] Comellas-Aragones M., Engelkamp H., Claessen V.I., Sommerdijk N.A., Rowan A.E., Christianen P.C., Maan J.C., Verduin B.J., Cornelissen J.J., Nolte R.J. (2007). A virus-based single-enzyme nanoreactor. Nat. Nanotechnol..

[60] Zakeri B. (2015). Synthetic Biology: A New Tool for the Trade. ChemBioChem.

[61] Fiedler J.D., Brown S.D., Lau J.L., Finn M.G. (2010). RNA-directed packaging of enzymes within virus-like particles. Angew. Chem..

[62] Uchida M., McCoy K., Fukuto M., Yang L., Yoshimura H., Miettinen H.M., LaFrance B., Patterson D.P., Schwarz B., Karty J.A. (2018). Modular Self-Assembly of Protein Cage Lattices for Multistep Catalysis. ACS Nano.

[63] Jordan P.C., Patterson D.P., Saboda K.N., Edwards E.J., Miettinen H.M., Basu G., Thielges M.C., Douglas T. (2016). Self-assembling biomolecular catalysts for hydrogen production. Nat. Chem..

[64] Patterson D.P., Schwarz B., El-Boubbou K., van der Oost J., Prevelige P.E., Douglas T. (2012). Virus-like particle nanoreactors: Programmed encapsulation of the thermostable CelB glycosidase inside the P22 capsid. Soft Matter.

[65] Schoonen L., Nolte R.J., van Hest J.C. (2016). Highly efficient enzyme encapsulation in a protein nanocage: Towards enzyme catalysis in a cellular nanocompartment mimic. Nanoscale.

[66] Brasch M., Putri R.M., de Ruiter M.V., Luque D., Koay M.S., Caston J.R., Cornelissen J.J. (2017). Assembling Enzymatic Cascade Pathways inside Virus-Based Nanocages Using Dual-Tasking Nucleic Acid Tags. J. Am. Chem. Soc..

[67] Minten I.J., Hendriks L.J., Nolte R.J., Cornelissen J.J. (2009). Controlled encapsulation of multiple proteins in virus capsids. J. Am. Chem. Soc..

[68] Rurup W.F., Verbij F., Koay M.S., Blum C., Subramaniam V., Cornelissen J.J. (2014). Predicting the loading of virus-like particles with fluorescent proteins. Biomacromolecules.

[69] Minten I.J., Claessen V.I., Blank K., Rowan A.E., Nolte R.J.M., Cornelissen J.J.L.M. (2011). Catalytic capsids: The art of confinement. Chem. Sci..

[70] Schoonen L., Pille J., Borrmann A., Nolte R.J., van Hest J.C. (2015). Sortase A-Mediated N-Terminal Modification of Cowpea Chlorotic Mottle Virus for Highly Efficient Cargo Loading. Bioconj. Chem..

[71] Patterson D.P., Schwarz B., Waters R.S., Gedeon T., Douglas T. (2014). Encapsulation of an enzyme cascade within the bacteriophage P22 virus-like particle. ACS Chem. Biol..

[72] Thuman-Commike P.A., Greene B., Jakana J., Prasad B.V., King J., Prevelige P.E., Chiu W. (1996). Three-dimensional structure of scaffolding-containing phage p22 procapsids by electron cryo-microscopy. J. Mol. Biol..

[73] Jiang W., Li Z., Zhang Z., Baker M.L., Prevelige P.E., Chiu W. (2003). Coat protein fold and maturation transition of bacteriophage P22 seen at subnanometer resolutions. Nat. Struct. Biol..

[74] Witherell G.W., Uhlenbeck O.C. (1989). Specific RNA binding by Q beta coat protein. Biochemistry.

[75] Giessen T.W., Silver P.A. (2016). A Catalytic Nanoreactor Based on in Vivo Encapsulation of Multiple Enzymes in an Engineered Protein Nanocompartment. ChemBioChem.

[76] Lau Y.H., Giessen T.W., Altenburg W.J., Silver P.A. (2018). Prokaryotic nanocompartments form synthetic organelles in a eukaryote. Nat. Commun..

[77] Sigmund F., Massner C., Erdmann P., Stelzl A., Rolbieski H., Desai M., Bricault S., Worner T.P., Snijder J., Geerlof A. (2018). Bacterial encapsulins as orthogonal compartments for mammalian cell engineering. Nat. Commun..

[78] Bae Y., Kim G.J., Kim H., Park S.G., Jung H.S., Kang S. (2018). Engineering Tunable Dual Functional Protein Cage Nanoparticles Using Bacterial Superglue. Biomacromolecules.

[79] Duda R.L., Oh B., Hendrix R.W. (2013). Functional domains of the HK97 capsid maturation protease and the mechanisms of protein encapsidation. J. Mol. Biol..

[80] Corchero J.L., Cedano J. (2011). Self-assembling, protein-based intracellular bacterial organelles: Emerging vehicles for encapsulating, targeting and delivering therapeutical cargoes. Microb. Cell Fact..

[81] Williams E.M., Jung S.M., Coffman J.L., Lutz S. (2018). Pore Engineering for Enhanced Mass Transport in Encapsulin Nanocompartments. ACS Synth. Biol..

[82] Rurup W.F., Snijder J., Koay M.S., Heck A.J., Cornelissen J.J. (2014). Self-sorting of foreign proteins in a bacterial nanocompartment. J. Am. Chem. Soc..

[83] Cassidy-Amstutz C., Oltrogge L., Going C.C., Lee A., Teng P., Quintanilla D., East-Seletsky A., Williams E.R., Savage D.F. (2016). Identification of a Minimal Peptide Tag for in Vivo and in Vitro Loading of Encapsulin. Biochemistry.

[84] Rurup W.F., Cornelissen J.J., Koay M.S. (2015). Recombinant expression and purification of “virus-like” bacterial encapsulin protein cages. Methods Mol. Biol..

[85] Zeth K., Hoiczyk E., Okuda M. (2016). Ferroxidase-Mediated Iron Oxide Biomineralization: Novel Pathways to Multifunctional Nanoparticles. Trends Biochem. Sci..

[86] Giessen T.W., Silver P.A. (2016). Converting a Natural Protein Compartment into a Nanofactory for the Size-Constrained Synthesis of Antimicrobial Silver Nanoparticles. ACS Synth. Biol..

[87] Snijder J., Kononova O., Barbu I.M., Uetrecht C., Rurup W.F., Burnley R.J., Koay M.S., Cornelissen J.J., Roos W.H., Barsegov V. (2016). Assembly and Mechanical Properties of the Cargo-Free and Cargo-Loaded Bacterial Nanocompartment Encapsulin. Biomacromolecules.

[88] Putri R.M., Allende-Ballestero C., Luque D., Klem R., Rousou K.A., Liu A., Traulsen C.H., Rurup W.F., Koay M.S.T., Caston J.R. (2017). Structural Characterization of Native and Modified Encapsulins as Nanoplatforms for in Vitro Catalysis and Cellular Uptake. ACS Nano.

[89] Chevalier A., Silva D.A., Rocklin G.J., Hicks D.R., Vergara R., Murapa P., Bernard S.M., Zhang L., Lam K.H., Yao G. (2017). Massively parallel de novo protein design for targeted therapeutics. Nature.

[90] Dang B., Wu H., Mulligan V.K., Mravic M., Wu Y., Lemmin T., Ford A., Silva D.A., Baker D., DeGrado W.F. (2017). De novo design of covalently constrained mesosize protein scaffolds with unique tertiary structures. Proc. Natl. Acad. Sci. USA.

[91] Dou J., Vorobieva A.A., Sheffler W., Doyle L.A., Park H., Bick M.J., Mao B., Foight G.W., Lee M.Y., Gagnon L.A. (2018). De novo design of a fluorescence-activating beta-barrel. Nature.

[92] Marcos E., Chidyausiku T.M., McShan A.C., Evangelidis T., Nerli S., Carter L., Nivon L.G., Davis A., Oberdorfer G., Tripsianes K. (2018). De novo design of a non-local beta-sheet protein with high stability and accuracy. Nat. Struct. Mol. Biol..

[93] Shen H., Fallas J.A., Lynch E., Sheffler W., Parry B., Jannetty N., Decarreau J., Wagenbach M., Vicente J.J., Chen J. (2018). De novo design of self-assembling helical protein filaments. Science.

[94] Huang P.S., Boyken S.E., Baker D. (2016). The coming of age of de novo protein design. Nature.

[95] Li H., Zheng G., Zhu S. (2018). Construction of an organelle-like nanodevice via supramolecular self-assembly for robust biocatalysts. Microb. Cell Fact..

[96] Zhu S., Zheng G. (2018). Dynamic kinetic resolution of Vince lactam catalyzed by gamma-lactamases: A mini-review. J. Ind. Microbiol. Biotechnol..

[97] Assaf Z., Faber K., Hall M. (2016). Scope, limitations and classification of lactamases. J. Biotechnol..

[98] Giessen T.W., Silver P.A. (2017). Engineering carbon fixation with artificial protein organelles. Curr. Opin. Biotechnol..

